# Effects of Goal-Framed Messages on Mental Health Education Among Medical University Students: Moderating Role of Personal Involvement

**DOI:** 10.3389/fpubh.2019.00371

**Published:** 2019-12-17

**Authors:** Li Bai, Qingmao Rao, Zhengjie Cai, Yalan Lv, Tingting Wu, Zumin Shi, Manoj Sharma, Yong Zhao, Xiaorong Hou

**Affiliations:** ^1^College of Medical and Informatics, Chongqing Medical University, Chongqing, China; ^2^Hospital of Zigong Mental Health Central, Zigong, China; ^3^School of Public Health and Management, Chongqing Medical University, Chongqing, China; ^4^Human Nutrition Department, Qatar University, Doha, Qatar; ^5^Behavioral & Environmental Health, School of Public Health, Jackson State University, Jackson, MS, United States; ^6^Chongqing Engineering Research Center for Clinical Big-Data and Drug Evaluation, Chongqing, China

**Keywords:** goal-framed message, personal involvement, mental health education, medical university students, prospect theory, gain- and loss-framed message

## Abstract

Mental health problem among university students is an emerging public health issue, and mental health education has always been the focus of attention for universities. However, limited attention has been paid to the effect of students' acceptance of health messages. Previous studies have found that message framing plays a key role in the process of responding to health-promoting messages. In this backdrop, the study aimed to examine the effects of goal-framed messages on mental health education among medical university students and investigate the moderating role of personal involvement. A cross-sectional study was conducted on medical university students. An online self-administered questionnaire was used to collect data. Wilcoxon rank-sum test and ordinal logistic regression were used for data analysis. Results showed significant differences in message acceptance between the gain- and loss-framed groups (*p* < 0.001). Participants with high personal involvement had higher message acceptance than those with low personal involvement in gain- and loss-framed message models (*p* < 0.05). Specifically, participants who related to roommates with high intimacy had higher message acceptance than those who related to roommates generally (*p* < 0.05). Participants who were concerned about their health condition had higher message acceptance than those who were neutral about their health condition (*p* < 0.001). Evidence of advantages of gain- over loss-framed messages on mental health among medical university students was found. The hypothesis that personal involvement with a health issue affects the acceptance of message framing was supported. Public health advocates can use framed message as a strategy to improve the efficacy of intervention in mental health education.

## Introduction

Mental health continues to be a critical component of public health. Mental health problem among students in higher education is an emerging public health issue (1). Mental distress and health problems have substantial and negative impacts on tertiary students' academic performance. A previous study has shown that psychological morbidity is an important reason for temporary or permanent withdrawal of students from courses (2). Globally, the prevalence of mental health problems and distress is prominent among tertiary students and is similar to or greater than those of the general population in each country (3). Mental health problems are increasing rapidly, constitute a significant health concern, and have been commonly reported among medical students (4, 5). Medical programs have always been considered a popular choice in higher education but have great academic pressure (4). Medical programs are competitive and stressful, which could cause adverse effects on mental health and lead to psychological disorders (4). Importantly, medical students are future doctors, who are the caregivers for the physical and mental well-being of patients, which is why focus on their mental health education is essential (6). Thus, essential and valid mental health education is valuable to medical university students.

However, promotion of mental health or preventive interventions of mental ill health for students have certain limitations (7). Early meta-analysis has revealed that no superiority of intervention group appears among studies with psychology educational interventions (1, 2). The results showed that effective help seeking remains low (8), and inadequate mental health knowledge is common among students (9). Therefore, public health advocates or professionals should seek an effective health communication strategy to motivate people to modify unhealthy behaviors or adopt healthy ones.

Health behaviors involve cognition processes related to motivation and decision making. Health care researchers need to explore empirically validated methods of motivating the public to comply with health recommendations. One such cognitively centered approach involves manipulating the phrasing or “framing” of health recommendations (10).

Principles in persuasive communication science offer useful message framing strategies that can enable favorable attention processes and achieve other persuasive outcomes. Goal framing research involves persuasive communication messages that generally examine how people's judgments of a single message-advocated object, position, or act may differ as a function of how the message is framed (11). Some persuasive health appeals focus on the benefits and positive consequences of taking a recommended behavior; nevertheless, others stress the costs and negative consequences of not adopting the recommendation. For example, health messages can highlight either the benefits of engaging in promoted health behavior (gain) or the negative consequences of not engaging in health behavior (loss) (12). Rothman and Salovey proposed that people will have high acceptance of loss-framed message when the outcome of the decision to engage in a health behavior involves some degree of uncertainty or risk; meanwhile, they will have high acceptance of gain-framed message when the outcome is certain or safe (13). Consistent with the deduction, Rothman and Salovey proposed that gain-framed message will be effective in promoting behaviors of prevention; meanwhile, loss-framed message will be effective in promoting behavior of illness detection (13). The distinction between prevention and detection behavior has been verified to be a useful heuristic for understanding the impact of framed message; the pattern of findings across studies is consistent (14).

However, some unresolved issues exist. Several recent meta-analyses (15, 16) do not confirm a persuasive advantage for gain- or loss-framing strategies for a range of health behaviors, which causes uncertainty in the gain- and loss-framing taxonomy. To probe the reasons for these inconsistent results, researchers should investigate under which circumstances gain- or loss-framed message is persuasive, which stresses the importance of exploring the moderating role. Investigation of potential moderating variables that can explain differences in the effects of gain- and loss-framed messages is indispensable to enhance our understanding of the effects of message framing on persuasion (17). Several possible moderator variables of message framing effects have been observed, such as heuristic–systematic model (HSM) (11, 18), self-efficacy (19), and risk perceptions (20). However, a study of framed message and mammography demonstrated no differences in self-efficacy between models who received either a gain-framed or a loss-framed video (21). Previous studies have demonstrated that HSM and risk perceptions are related to information processing (22, 23).

Personal involvement of the goal-framed message for the recipient is a factor that has not received considerable attention to date. A previous study has argued that a message should be integrated into the individual's cognitive representation of the issue to ensure its influence on behaviors (13). This integration is presumably achieved through systematic processing, which is motivated by issue involvement being targeted (17, 24). Personal involvement has been shown to play an instrumental role in the way that people respond to and process health recommendation messages (17). Studies have explored the effect of personal involvement on message framing. Millar and Millar investigated the effect of framing and issue involvement on the intentions of participants to perform a safe driving behavior (25). They demonstrated that gain-framed messages are more effective than loss-framed messages, and participants with high issue-involvement conditions produce higher cognition than participants with low issue-involvement conditions (25). We previously examined the acceptance of different modes of food safety framed message (26). We found that loss-framed messages receive higher acceptance than gain-framed messages, and participants in the high level of personal involvement exhibit high acceptance (26). However, only one study has examined which message frame (gain, loss, or neutral) of appointment reminder letters is associated with attendance rates in specialty mental health appointments; the results showed that participants who received letters with gain-framed messages attend their scheduled appointments at a higher rate than those who received a routine letter with no additional message (27).

Several studies have concluded that framed effect may be used when people are concerned or highly educated regarding health issues (25, 26, 28). Previous studies also have found that being in love before (29) and serving as a student cadre (30) can affect student's mental health. Several studies have also demonstrated that lack of siblings (31), relationship with roommates (32), encountering insomnia (33), concerning health condition (34), emotional management (35), and encountering anxiety (36) are related to student's mental health. With this backdrop, the present study aimed to explore the effects of goal-framed messages on the mental health education among medical university students in China and examine the moderating role of personal involvement.

## Materials and Methods

### Participants

The study used a stratified cluster random sampling method to select participants. A total of 1,245 medical students were included in this analysis. A total of 490 males and 755 females were included. Freshmen medical students were 221 [17.8%], sophomores were 743 (59.7%), and juniors were 281 (22.6%). A total of 629 (50.5%) participants were from urban areas. Minority respondents constituted 10.6%. The monthly living expenses of 55.1% of the participants ranged from 1,000–1,499 CNY.

### Stimulus Materials

The questionnaire was initially developed on the basis of an extensive review of mental health studies (37–39) and finalized after an expert panel reached a consensus. Eight items were used to estimate the degree of personal involvement. Message framing materials were grouped into four sections, namely, academic, interpersonal relationships, self-rating, and career that related to undergraduate students closely on the basis of previous literature (1, 40).

### Procedures

The final data were collected from an online survey conducted in a computer laboratory on campus. All investigators of this study were recruited through interview to join the investigation team. Participants filled out questionnaires online in a laboratory. During the survey, the researchers were present and answered questions raised by the participants. Confidentiality of all of the collected data was maintained throughout the study. All subjects signed their informed consent for inclusion before participating.

The study was carried out in accordance with the recommendations of the Ethics Committee of Chongqing Medical University. The protocol was approved by the Ethics Committee of Chongqing Medical University (Approval number 2018011).

### Measures

#### Personal Involvement Types

Personal involvement is the extent which message recipients perceived the object or objective to be self-related or in some way instrumental in achieving their personal value and goal (28). The use of personal involvement variables was based on the following literatures. (1) Love experience and love status affect the obvious symptoms (e.g., depression, anxiety) in the mental health of college students (29). (2) Student cadres of ethnic universities are under heavy pressure of work with less time for study and many mental burdens, mental problems cannot be solved in time, and they often have many interpersonal obstacles (30). (3) Mental health status of the only child is significantly worse than that of the non-only child (31). (4) Roommate relationships on college student mental health—the roommate relationship is a specific interpersonal system that affects students' mental health and ability to cope with university life (32). (5) Many individuals with mental disorders also report insomnia (33). (6) Individuals with high health consciousness showed a high level of fear as well as anxiety, and health consciousness is likely to lead people to take responsibilities for their own health, which ultimately results in having high self-efficacy to overcome mental illness (34). (7) Psychological health is an important determinant of the individual development of college students, and emotional management is particularly important in the mental health of college students (35). (8) Academic anxiety is significantly but negatively correlated with mental health for both male and female adolescents (36).

In addition, these personal involvement variables were assigned value completed after several discussions by a panel of experts. (1) Have you been in love before? (1 = no and 2 = yes); (2) Did you serve as a student cadre? (1 = no and 2 = yes); (3) Lack of siblings? (1 = no and 2 = yes); (4) Hhow do you feel about the relationships between you and your roommates? (1 = very indifferent, 2 = indifferent, 3 = general, 4 = intimate, and 5 = very intimate); (5) Did you have symptoms of insomnia? (1 = never, 2 = occasionally, and 3 = often); (6) To what degree are you concerned about your health condition? (1 = not concerned, 2 = general, and 3 = concerned); (7) How do you feel about your emotional control? (1 = very good, 2 = good, 3 = general, 4 = bad, and 5 = very bad); and (8) Did you encounter emotional anxiety? (1 = never, 2 = occasionally, and 3 = often).

#### Message Framing Materials

The framed persuasive messages comprised a short message leading to either positive or negative consequences. Gain-framed message highlights the benefits of engaging in health behavior (“If you join the ‘team learning,' then positive learning attitude and methods may be improved.”). By contrast, loss-framed message emphasizes the costs of not engaging in health behavior (“If you do not join the ‘team learning,' then positive learning attitude and methods may be not improved.”). A corresponding image was attached to each message to improve their persuasive effects. A 5-point Likert scale ranging from strongly disagree to strongly agree (1 = “totally disagree” to 5 = “totally agree”) was utilized to assess the extent of their agreement, as shown in Table 1.

**Table 1:** Message framing materials.

**Message framing materials**		**Gain-framed**	**Loss-framed**
Academic	Anxious	If you are properly anxious, the ability of learning may be improved.	If you are constantly anxious, the ability of learning may be reduced.
	Team-learning	If you join the “team learning,” then positive learning attitude and methods may be improved.	If you do not join the “team learning,” then positive learning attitude and methods may be not improved.
Interpersonal- relationships	Listening to friends	If you listen to friends with patience, the friendship may be more intimate.	If you listen to friends without patience, your friends may distance themselves from you.
	Online communication	If you use online communication in moderation, the risk of mental disorders (depression, anxiety, etc.) may be reduced.	If you are addicted to online communication, the risk of mental disorders (depression, anxiety, etc.) may be increased.
	Joining in the clubs	If you join “clubs,” interpersonal skill may be improved.	If you do not join “clubs,” interpersonal skill may be obstructed.
Self-rating	Inferiority	If you overcome inferiority, you may promote personality self-development.	If you develop a sense of inferiority, you may hinder personality self-development.
	Arrogance	If you overcome arrogance, you may maintain a favorable interpersonal relationship.	If you develop a sense of arrogance, you may maintain an unfavorable interpersonal relationship.
	Self-rating objectively	If you recognize or assess yourself objectively, you may succeed more easily.	If you misjudge yourself, you may succeed more uneasily.
Career	Following the crowd blindly	If you do not follow the crowd blindly when choosing a job, you may find an appropriate job quickly.	If you follow the crowd blindly when choosing a job, you may fail to find an appropriate job quickly.
	Dependent character	If you develop an independent character, you may grow quickly in career.	If you form the habit of dependence on others, you may grow slowly in career.

The Cronbach's alpha coefficients were 0.88, 0.899, and 0.863 for the entire questionnaire (personal involvement variables and message framing materials), gain-framed message materials, and loss-framed message materials, respectively.

Moreover, the order of the message was varied across the frame by a random number table to disguise message manipulation. The framed message materials in the study were designed on the basis of the language and approaches used in the previous studies.

### Manipulation Check

#### Personal Involvement Type

The scores of all of the personal involvement variables of this study were first summed and then categorized into two groups. The score of personal involvement about participants ranged from 10 to 22 units. The number of participants who had a score between 18 and 22 units was 623 (50%), which were defined as high-personal involvement group.

#### Message Framing

The framed messages were tested by Wilcoxon signed rank-sum test. The acceptance of message framing was expressed as the median, and high scores indicated high message acceptance. The study counted the scores of all message framing item groups by different frames. The scores were categorized into tertiles. Ordinal logistic regression analysis was implemented to analyze the factors associated with framing effects of mental health. In the ordinal logistic regression model, the independent variables included “gender,” “grade,” “residence,” “ethnicity,” “monthly living expenses,” “lack of siblings,” “have you been in love before,” “did you serve as a student cadre,” “how do you feel about the relationships between you and your roommates,” “did you have symptoms of insomnia,” “to what degree are you concerned about your health condition,” “how do you feel about your emotional control,” and “how often did you have emotional anxiety.”

## Results

### Testing Framing Effects

Table 2 shows that many gain- and loss-framed mental health messages had significant differences (*p* < 0.05) among subjects: (1) anxious; (2) team learning; (3) listening to friends; (4) online communication; (5) joining in the clubs; (6) inferiority; (7) self-rating objectively; and (8) following the crowd blindly. Nevertheless, two sets of messages showed inconsistencies. No significant difference was observed for messages of arrogance and dependent character (*p* > 0.05).

**Table 2:** Analyses of the gain- and loss-framed mental health messages.

**Variables**	**Gain-framed**					**Loss-framed**					***p***
	***n*** **(%)**					***n*** **(%)**					
	**1**	**2**	**3**	**4**	**5**	**1**	**2**	**3**	**4**	**5**	
Anxious	7 (0.6%)	13 (1%)	89 (7.1%)	628 (50.4%)	508 (40.8%)	0 (0%)	4 (0.3%)	50 (4%)	495 (39.8%)	696 (55.9%)	0.000^**^
Team-learning	2 (0.2%)	8 (0.6%)	88 (7.1%)	540 (43.4%)	607 (48.8%)	3 (0.2%)	28 (2.2%)	146 (11.7%)	559 (44.9%)	509 (40.9%)	0.000^**^
Listening to friends	0 (0%)	3 (0.2%)	58 (4.7%)	538 (43.2%)	646 (51.9%)	4 (0.3%)	18 (1.4%)	89 (7.1%)	534 (42.9%)	600 (48.2%)	0.000^**^
Online communication	1 (0.1%)	3 (0.2%)	47 (3.8%)	530 (42.6%)	664 (53.3%)	23 (1.8%)	112 (9%)	344 (27.6%)	538 (43.2%)	228 (18.3%)	0.000^**^
Joining in the clubs	1 (0.1%)	8 (0.6%)	117 (9.4%)	552 (44.3%)	567 (45.5%)	10 (0.8%)	22 (1.8%)	163 (13.1%)	559 (44.9%)	491 (39.4%)	0.000^**^
Inferiority	1 (0.1%)	5 (0.4%)	48 (3.9%)	506 (40.6%)	685 (55%)	1 (0.1%)	7 (0.6%)	70 (5.6%)	585 (47%)	582 (46%)	0.000^**^
Arrogance	2 (0.2%)	2 (0.2%)	57 (4.6%)	514 (41.3%)	670 (53.8%)	2 (0.2%)	3 (0.2%)	63 (5.1%)	515 (41.4%)	662 (53.2%)	0.431
Self-rating objectively	1 (0.1%)	3 (0.2%)	49 (3.9%)	521 (41.8%)	671 (53.9%)	1 (0.1%)	6 (0.5%)	63 (5.1%)	554 (44.5%)	621 (49.9%)	0.000^**^
Following the crowd blindly	4 (0.3%)	12 (1%)	172 (13.8%)	581 (46.7%)	476 (38.2%)	1 (0.1%)	1 (0.1%)	64 (5.1%)	544 (43.7%)	635 (51%)	0.000^**^
Dependent character	3 (0.2%)	7 (0.6%)	66 (5.3%)	519 (41.7%)	650 (52.2%)	2 (0.2%)	3 (0.2%)	59 (4.7%)	556 (44.7%)	625 (50.2%)	0.756

*1, totally disagree; 2, disagree; 3, general; 4, agree; 5, totally agree*.

** *p < 0.001 (statistically significant)*.

Table 3 shows a significant difference between gain- and loss-framed messages (*p* < 0.001). Higher acceptance on the gain-framed messages than loss-framed message among participants was observed (*p* < 0.001).

**Table 3:** Analyses of the gain- and loss-framed mental health messages by personal involvement.

	**Gain-framed**	**Loss-framed**		
	**Median**		**Z**	***p***
Low personal involvement	43	42	−9.52	0.000^**^
High personal involvement	45	44	−10.167	0.000^**^
Total	44	43	−13.87	0.000^**^

** *p < 0.001 (statistically significant)*.

### Factors Associated With the Message Acceptance

Table 4 shows that in the gain-framed message model, subjects who related to roommates generally were less likely to get high message acceptance [odds ratio (OR) = 0.631, 95% confidence interval (CI) 0.439–0.906, *p* < 0.05] than those who related to roommates with high intimacy. Subjects who were neutral regarding their health condition were less likely to gain high message acceptance (OR = 0.656, 95% CI 0.528–0.814, *p* < 0.001) than those who were concerned about their health condition. In the loss-framed message model, males were less likely to get high message acceptance (OR = 0.741, 95% CI 0.594–0.925, *p* < 0.05) than females. Participants who related to roommates with intimacy were less likely to get high message acceptance (OR = 0.736, 95% CI 0.557–0.972, *p* < 0.05) than those who related to roommates with high intimacy. Subjects who were neutral regarding their health condition were less likely to get high message acceptance (OR = 0.682, 95% CI 0.555–0.846, *P* < 0.001) than those who were concerned about their health condition.

**Table 4:** Ordinal logistic regression for the factor effects of the message-framing acceptance.

**Parameter**		**Gain-framed message**		**Loss-framed message**	
		**OR (95% CI)**	***p***	**OR (95% CI)**	***p***
Intercept1		1.001 (0.12–8.335)	0.999	0.723 (0.089–5.856)	0.761
Intercept2		4.256 (0.511–35.483)	0.181	3.365 (0.415–27.281)	0.256
Gender	Male	0.888 (0.711–1.107)	0.291	0.741 (0.594–0.925)	0.008^*^
	Female(ref.)				
Grade	Freshman	0.868 (0.619–1.218)	0.398	1.046 (0.75–1.458)	0.791
	Sophomore	1.033(0.798–1.339)	0.804	1.133 (0.875–1.466)	0.344
	Junior(ref.)				
Current residence	Urban	0.963 (0.763–1.216)	0.751	0.925 (0.733–1.168)	0.513
	Rural(ref.)				
Ethnicity	Han nationality	0.968 (0.685–1.368)	0.853	0.876 (0.614–1.225)	0.419
	Minority(ref.)				
Monthly living expenses	< ¥1,000	2.104(0.87–5.090)	0.099	1.715 (0.708–4.157)	0.232
	¥1,000–¥1,499	1.769 (0.749–4.177)	0.193	1.63 (0.689–3.857)	0.266
	¥1,500–¥1,999	1.884 (0.782–4.535)	0.158	1.572 (0.652–3.793)	0.314
	¥2,000– ¥2,499	2.368 (0.886–6.333)	0.086	1.824 (0.684–4.864)	0.23
	>¥2,500(ref.)				
lack of siblings	No	0.822 (0.649–1.041)	0.104	0.883 (0.698–1.117)	0.299
	Yes(ref.)				
Have you been in love before	No	0.949 (0.767–1.175)	0.632	0.908 (0.735–1.123)	0.374
	Yes(ref.)				
Did you serve as a student cadre	No	1.063 (0.823–1.373)	0.641	1.163 (0.901–1.50)	0.246
	Yes(ref.)				
How do you feel about the relationships between you and your roommates	Very indifferent	0.296 (0.07–1.255)	0.099	0.366 (0.086–1.55)	0.172
	Indifferent	1.426 (0.58–3.506)	0.44	1.486 (0.614–3.597)	0.38
	General	0.631 (0.439–0.906)	0.013^*^	0.712 (0.496–1.02)	0.064
	Intimate	0.772 (0.583–1.023)	0.072	0.736 (0.557–0.972)	0.031^*^
	Very intimate(ref.)				
Did you have symptoms of insomnia	Never	1.09 (0.671–1.769)	0.729	0.873 (0.538–1.415)	0.581
	Occasionally	1.043 (0.678–1.604)	0.847	0.803 (0.523–1.233)	0.316
	Often(ref.)				
To what degree are you concerned about your health condition	Not concerned	0.503 (0.234–1.08)	0.078	0.599 (0.279–1.285)	0.188
	General	0.656 (0.528–0.814)	0.000^**^	0.682 (0.555–0.846)	0.001^**^
	Concerned(ref.)				
How do you feel about your emotion controlling	Very good	3.908 (0.567–26.943)	0.166	2.791 (0.417–18.678)	0.29
	Good	2.794 (0.413–18.913)	0.292	2.322 (0.353–15.284)	0.281
	General	2.535 (0.373–17.219)	0.341	2.453(0.372–16.197)	0.351
	Bad	3.638 (0373–18.673)	0.331	2.306 (0.335–15.88)	0.396
	Very bad(ref.)				
How often did you have emotional anxiety	Never	1.134 (0.602–2.134)	0.697	1.046 (0.562–1.948)	0.888
	Occasionally	0.855 (0.603–1.211)	0.377	0.902 (0.637–1.277)	0.563
	Often(ref.)				

* p < 0.05 and

** *p < 0.001 (statistically significant)*.

## Discussion

The study examined the effects of goal-framed messages on mental health among medical university students in Chongqing, China, and explored the moderating role of personal involvement. A significant difference was observed between gain- and loss-framed mental health messages in the present cross-sectional study. Meanwhile, a significant difference was found between participants in the higher personal involvement and participants in the lower personal involvement. These results are intriguing on multiple levels. First, the finding has implications for the manner and tone of mental health advocates or professionals. The finding here suggested that emphasizing the positive effects or gain-framed messages of mental health education may be more effective than underscoring the negative effects or loss-framed messages. Participants exposed to gain-framed message showed higher acceptance than those who exposed to loss-framed message. Second, this study also expressed support for the contention that personal involvement may moderate the acceptance of message framing. Participants with a high level of personal involvement showed higher acceptance of mental health message than those with a low level of personal involvement.

Consistent with previous literature, our findings demonstrated that gain-framed messages exhibited to be more effective than loss-framed messages in enhancing mental health education. Rothman and Salovey applied prospect theory to determine the response of people to health-framed messages and proposed that the behavior function can suggest how risky people are likely to view performing the behavior (13). Behavior that serves an illness prevention function (i.e., physical activity) should often be viewed as involving little risk, and only the risk is not engaging in these behaviors. Meanwhile, behavior that serves an illness detection function (i.e., mammography) should be likely to be viewed as involving a high degree of risk because of the possibility that a serious illness can be discovered (13). From the point of view, Rothman and Salovey argued that the underlying function of a health behavior should serve as a useful heuristic for the perceived riskiness of health behavior and should moderate people's responses to framed messages (13). Particularly, they suggested that gain-framed message would be persuasive for disease prevention behavior, and loss-framed message would be persuasive for disease detection behavior (16). Previous research has demonstrated similar results (41, 42). Our study also demonstrated notable framing effects of illness prevention behaviors, including (1) team leaning, (2) listening to friends, (3) online communication, (4) joining in the clubs, (5) inferiority, (6) self-rating objectively, and (7) dependent character.

However, a few messages, such as (1) being anxious, (2) having arrogance, and (3) following the crowd blindly, elicited higher message acceptance using loss-framed description than using gain-framed description in this study. The reason for these inconsistent results may be that such behaviors are considered high-risk behaviors or may lead to an uncertain result. This conclusion was similar to that of an empirical study, which revealed that participants responded favorably to gain-framed message when the risk associated with a health behavior (either a prevention behavior or a detection behavior) was low; by contrast, participants responded favorably to loss-framed message when the risk associated with the health behavior (either prevention or detection) was high (43). Considerable research evidence has confirmed that clinically anxious patients, who typically exhibited elevated levels of trait and state anxiety, are characterized by a processing bias that operates to favor selectively the encoding of emotionally threatening stimuli (44, 45). Numerous findings are in accordance with the view that arrogance is an undesirable virtue, which is considered a high-risk behavior that may lead to failure (46, 47). Finally, “following the crowd blindly” is related to seeking a job in the future, which is an uncertain result. This result is also consistent with Rothman's perspective that people will show high acceptance of loss-framed message when the outcome of the decision to engage in a behavior involves some degree of uncertainty (13).

The effects of message framing on health behavior would be influenced by the person's level of involvement with a health issue (13). A framed message should be integrated into the individual's cognitive representation of the issue to ensure its effect on behaviors. Persuasive messages are believed to be processed either systematically (with attention to details) or heuristically (focusing on the overall impression), with great cognitive integration being achieved through systematic processing (24). Involvement, or interest in an issue, is presumed to motivate systematic processing, and framed message effects are likely to occur if the target audience has a high level of health issue involvement (24). Research has taken personal involvement as a moderating factor in message framing effects (25, 48).

Our findings showed that subjects with a high personal involvement had higher message acceptance than those with low personal involvement in gain- and loss-framed message models. Specifically, participants who related highly intimately with their roommates had higher message acceptance than those who only related generally with their roommates; participants who were concerned regarding their health condition had higher message acceptance than those who were neutral about their health condition. These conclusions were consistent with those in previous studies.

The study also had several limitations. First, owing to the cross-sectional study design, no causal inferences regarding the results can be made, and the findings may have a high risk of bias. Second, this study only investigated medical students. The findings cannot be generalized to all of the university students in China. Third, given the limitation of self-administered questionnaire design, the mental health construct may vary and can affect the study result. Hence, the results may not be generalized to other populations. Fourth, many other factors, such as drug use and family issues (broken families, abuse, etc.), were not included in this study. Further studies could include drug use and family issues. Finally, the study considered the effect of “grade” on the acceptance of mental health message and not included the effect of “age.” The “age” should be included in future studies. Despite these limitations, the study portrayed the selection preference of message framing on mental health education among medical students in China. This research also provided insight into the effects of personal involvement and framed message in the context of mental health education among medical university students in China.

## Conclusion

Consistent with prospect theory and previous research, this study found evidence of the advantages of gain-framed message over loss-framed message on mental health among medical university students in Chongqing, China. The present study also supported the hypothesis that personal involvement with a health issue affects the acceptance of message framing. Our finding demonstrated that participants with a high level of personal involvement showed high acceptance of mental health message. The findings of this research have a significant impact for the provision of public health information. Public health professionals and advocates can use message framing as a strategy to improve intervention efficacy in the process of mental health education among the future frontline mental health care of medical students.

## Data Availability Statement

All datasets generated for this study are included in the article/supplementary material.

## Ethics Statement

The studies involving human participants were reviewed and approved by Chongqing Medical University. The patients/participants provided their written informed consent to participate in this study.

## Author Contributions

XH, YZ, and LB designed and performed the experiments. ZC helped perform the experiments. LB wrote the paper and analyzed the data. YL helped analyze the data. ZC, TW, QR, YL, ZS, and MS helped draft the manuscript. All authors read and approved the final manuscript.

### Conflict of Interest

The authors declare that the research was conducted in the absence of any commercial or financial relationships that could be construed as a potential conflict of interest.
